# Synthesis of a New Chiral Pyrrolidine †

**DOI:** 10.3390/molecules15031501

**Published:** 2010-03-09

**Authors:** Mari Fe. Flores, Marta G. Núñez, Rosalina F. Moro, Narciso M. Garrido, Isidro S. Marcos, Enrique F. Iglesias, Pilar García, David Díez

**Affiliations:** Departamento de Química Orgánica, Universidad de Salamanca, Plaza de los Caídos 1-5, 37008, Salamanca, Spain

**Keywords:** organocatalysis, pyrrolidines, sulfones, nitrones

## Abstract

The synthesis of a new chiral pyrrolidine has been performed using 2,3-*O*-*iso*-propylidene-D-erythronolactol as a suitable starting material.

## 1. Introduction 

**Figure 1:**
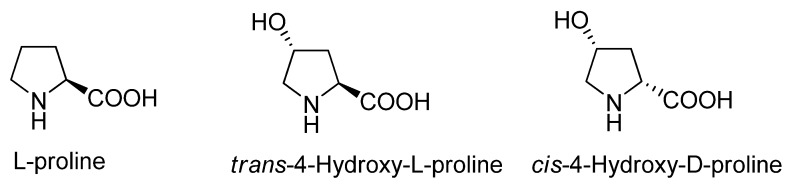
L-proline and two analogues.

In the last years there has been a growing interest in organocatalysis [1,2,3,4,5,6], a new field which has quickly attracted researchers’ attention due to its potential for saving costs, time and energy compared to classic catalysis. Among the many known organocatalysts L-proline is perhaps the one which has been most studied. This fact has led to the appearance of many analogues [7,8,9,10,11,12,13,14]. In the seminal paper of List, Lerner and Barbas III [15], it is described how in the aldol reaction the catalytic activity of L-proline increases using *trans*-4-hydroxy-L-proline and also how the enantiomeric excess reverses using *cis*-4-hydroxy-D-proline, (Figure 1).

**Scheme 1:**
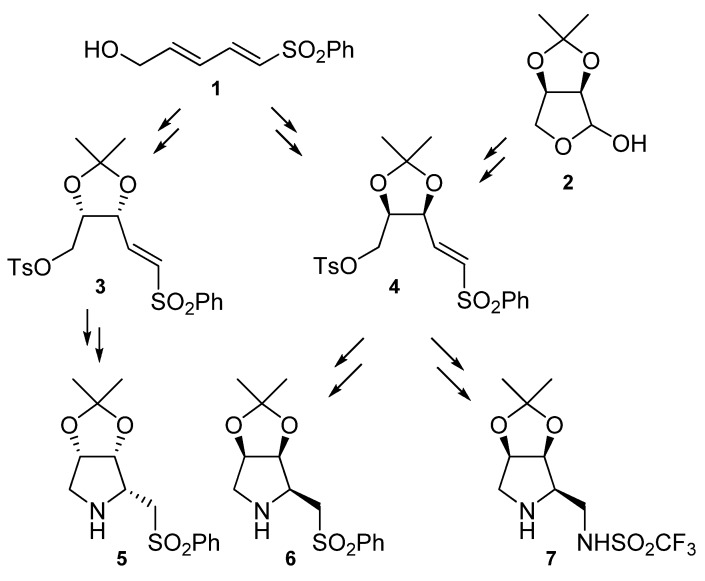
Synthesis of several pyrrolidines from a sulfonylbutadiene or 2,3-*O*-*iso*-propylidene-D-erythronolactol.

In our research group we have achieved the synthesis of several chiral pyrrolidines from sulfonylbutadiene **1** or 2,3-*O*-*iso*-propylidene-D-erythronolactol (**2**) (Scheme 1).

Starting from sulfonylbutadiene **1**, pyrrolidines **5** and **6** were obtained through vinyl sulfones **3** and **4**, respectively [16,17]. In order to increase the yields, a new route for the synthesis of compound **6** was devised starting from 2,3-*O*-*iso*-propylidene-D-erythronolactol (**2**) through vinylsulfone **4 [18]**. This pyrrolidine **6** has been proved to be an organocatalyst for the intramolecular *oxa*-Michael reaction [19,20]. Moreover, compound **7**, which has been reported to catalyze Michael reactions [21], has been synthesized from intermediate vinylsulfone **4** (Scheme 1). 

## 2. Results and Discussion

**Scheme 2:**
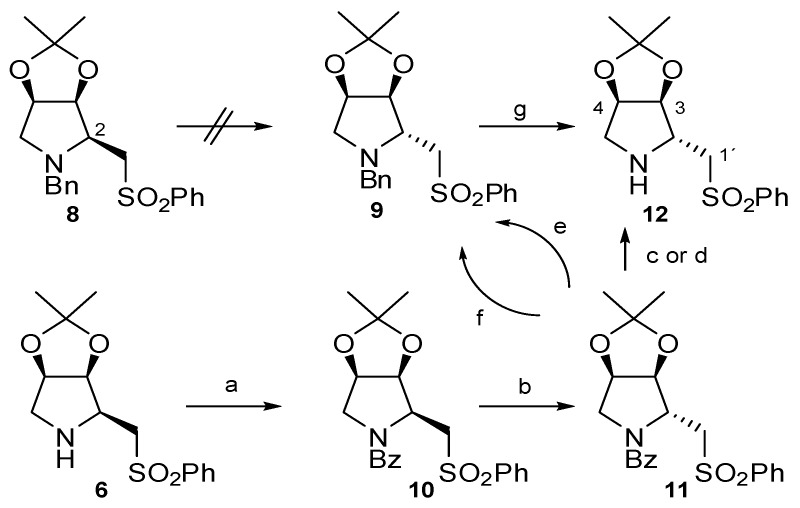
Epimerization of C-2 in compound **8**.

In this paper we describe our studies on the synthesis of the epimer at C-2 of pyrrolidine **6**, as we are interested in comparing the properties of both diastereoisomers in organocatalytic reactions. In previous studies with PPY-derivatives, we had observed the epimerization of that stereogenic center when it was treated with bases [22]. Taking this into account, we first tried the epimerization at C-2 in compound **8**, obtained directly from **4** by treatment with benzylamine. However, although several bases were used, none of them gave the epimerization. Therefore, we devised the following synthesis for the required compound **12** (Scheme 2).

Benzoylation of pyrrolidine **6** gave derivative **10** as outlined in Scheme 2. When this compound was submitted to treatment with *n*-BuLi, the C-2 epimer **11** was obtained in moderate yield. Once the required stereochemistry at C-2 in **11** was achieved, we proceeded to deprotect the nitrogen to obtain the desired pyrrolidine **12**. This step was not as simple as it was thought initially, since the desired direct debenzoylation did not take place under several conditions. Finally, it was necessary to reduce the benzoyl to benzyl group and then deprotect under the usual conditions. Although, the final deprotection took place in good yield, the previous transformation from benzoyl to benzylderivative was only achieved in low yield, making useless this procedure to synthesise **12**.

**Scheme 3:**
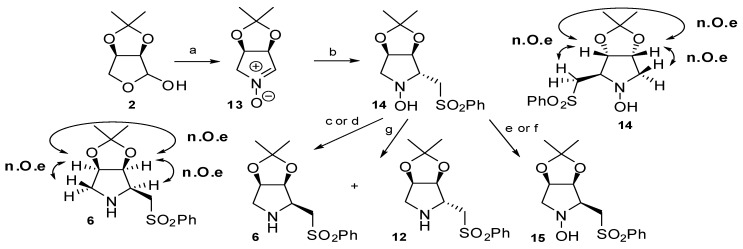
Synthesis of **12** from 2,3-*O*-*iso*-propylidene-D-erythronolactol.

Therefore, we devised a new synthesis of compound **12** starting from 2,3-*O*-*iso*-propylidene-D-erythronolactol (**2**). Goti [23,24,25] and Wightman and Closa [26,27,28] have obtained nitrone **13** from compound **2**. Besides, Merino and Goti have applied this versatile nitrone to the synthesis of iminocyclitols, pyrrolizidines and indolizidines [29,30], observing that the addition of organometallics to **13** took place to give the *trans* compounds. With this procedure in mind, we were able to achieve the desired compound **12** in a simple manner, as depicted in Scheme 3.

When compound **13** was treated with lithio(phenylsulfonyl)methane, only hydroxylamine **14** was obtained stereoselectively in moderate yield. The stereochemistry of **14** was established studying its NMR spectra (whose assignment is given in the Experimental section) and by the observation of the nOes that this molecule displays (Scheme 3). The nOes of the enantiomer of compound **6** has already been reported by our research group [16]. Once this compound **14** was synthesized, different reduction conditions to obtain the pyrrolidine ring were tested.

Hydrogenation under different conditions either Pd(OH)_2_ or Pd on carbon only leads to the pyrrolidine **6**. When **14** was submitted to reduction with stoichiometric indium or with zinc, hydroxylamine **15** with inversion at C-2 was obtained. Thus, it was necessary to choose the adequate conditions to reduce the hydroxylamine to the required pyrrolidine **12** without epimerization at C-2. This was achieved using catalytic indium and stoichiometric Zn [29,30,31] (Table 1, entry 8).

Having obtained our desired compounds **6** and **12**, they were tested as organocatalysts in Michael addition reactions of cyclohexanone to nitrostyrene, as shown in Table 2.

As can be observed, both compounds **6** and **12** are organocatalysts. However, their behaviour is rather different depending on the solvent chosen to carry out the reaction. It is worthy mentioning that compound **12** lead to the opposite enantiomer of **6** in the Michael addition, as a result of the stereochemistry change in C-2 position. The absolute configuration of the addition product was established by comparison of the HPLC data with the ones reported by us [21] and others [32,33].

## 3. Conclusions

The synthesis of a new chiral pyrrolidine **12**, has been achieved from the same starting material, 2,3-*O*-*iso*-propylidene-D-erythronolactol through two different methodologies. In addition, the reduction of the chiral hydroxylamine into pyrrolidine has been studied under different conditions.

## 4. Experimental 

### 4.1. General

^1^H-NMR and ^13^C-NMR spectra were recorded in CDCl_3_ at 200 and 400 MHz (^1^H) or 50 and 100 MHz (^13^C) on Varian 200 VX and BRUKER DRX 400 instruments, respectively. Multiplicities were determined by DEPT experiments. IR spectra were registered using a BOMEM 100 FTIR spectrophotometer. Optical rotations were determined using a Perkin-Elmer 241 polarimeter in a 1 dm cell and are given in units of 10-1 deg cm^2^ g^−1^. Concentrations are quoted in g per 100mL. The electron impact (EI) mass spectra were run on a VG-TS 250 spectrometer using a 70 eV ionizing voltage. HRMS were recorded using a VG Platform (Fisons) spectrometer using Chemical Ionization (ammonia as gas) or Fast Atom Bombardment (FAB) techniques. Thin layer chromatography (tlc) was performed on aluminum sheets coated with 60 F254 silica. Sheets were visualized using iodine, UV light or 1% aqueous KMnO_4_ solution. Column chromatography (CC) was performed with Merck silica gel 60 (70–230 mesh). Solvents and reagents were generally distilled prior to use: dichloromethane (DCM) from KOH.

### 4.2. Preparation of (2S,3S,4R)-N-Benzoyl-2-phenylsulfonylmethyl-3,4-isopropylidenedioxypyrrolidine *(**10**)*

To a solution of pyrrolidine **6** (40 mg, 0.13 mmol) in pyridine (0.5 mL) at 0 °C was added PhCOCl (20 µL) and the mixture was stirred for 3 h. The reaction was quenched by the addition of water (0.2 mL) at 0 °C and then the mixture was extracted with EtOAc (3 × 10 mL). The combined organic layers were washed with aqueous solutions of CuSO_4_ (20%), NaHCO_3_ (5%) and brine. After drying (Na_2_SO_4_), filtering, and concentrating, the crude residue was purified by flash chromatography (silica gel, n-hexane/EtOAc 8:2) to give benzoyl derivative **10** (52 mg, 97%). IR (film) ν (cm^−1^) 3066, 2987, 2939, 1643, 1584, 1447, 1383, 1307, 1249, 1215, 1153, 1085; ^1^H-NMR (400 MHz) δ = 8.03–7.34 (m, 10H), 4.83 (t, *J* = 5.8 Hz, 1H), 4.66–4.61 (m, 2H), 3.98 (dd, *J* = 14.0 and 3.7 Hz, 1H), 3.84 (dd, *J* = 14.0 and 9.0 Hz, 1H), 3.59 (m, 2H), 1.54 (s, 3H), 1.32 (s, 3H); ^13^C-NMR (100 MHz) δ = 171.3, 139.7, 135.3, 133.6, 130.8, 129.1, 128.3, 128.1, 127.7, 113.1, 78.7, 77.5, 54.8, 54.5, 53.2, 27.1, 25.2; HRMS (ESI) C_21_H_24_NO_5_S requires (M+H^+^) 402.1369; found 402.1364. [α]_D_^20^ = −67.1 (*c* 1.4, CHCl_3_).

### 4.3. Preparation of (2R,3S,4R)-N-Benzoyl-2-phenylsulfonylmethyl-3,4-isopropylidenedioxypyrrolidine *(**11**)*

A solution of pyrrolidine **10** (40 mg, 0.10 mmol) in THF (1 mL) under argon at −78 °C, was added *n*-BuLi (75 µL, 1.6 M in hexanes). The resulting mixture was stirred for 3 h, allowing to warm to rt, whereupon the reaction was quenched with saturated aqueous solution of NH_4_Cl (0.2 mL) and extracted with EtOAc (3 × 10 mL). The combined organic layers were washed with brine, dried (Na_2_SO_4_), filtered, and concentrated. The resulting crude residue was purified by flash chromatography (silica gel, n-hexane/EtOAc 7:3) to obtain **11** (20 mg, 50%). IR (film) ν (cm^−1^) 3064, 2978, 2938, 1634, 1577, 1413, 1319, 1217, 1152, 1074, 1046, 858; ^1^H-NMR (400 MHz) δ = 7.98–7.38 (m, 10H), 5.25–5.23 (m, 1H), 4.85 (m, 1H), 4.79 (m, 1H), 3.89 (dd, *J* = 14.0 and 3.6 Hz, 1H), 3.78–3.71 (m, 2H), 3.44 (d, *J* = 14.0 Hz, 1H), 1.44 (s, 3H), 1.33 (s, 3H); ^13^C-NMR (100 MHz) δ = 170.0, 139.6, 135.5, 134.0, 130.2, 129.4, 128.3, 127.8, 127.2, 112.2, 82.5, 79.6, 60.2, 54.8, 54.7, 26.9, 24.9; HRMS (ESI) C_21_H_23_NO_5_S requires (M+Na) 424.1195; found 424.1189. [α]_D_^20^ = + 33.9 (*c* 1.1, CHCl_3_).

### 4.4. Preparation of (2R,3S,4R)-N-Benzyl-2-phenylsulfonylmethyl-3,4-isopropylidenedioxypyrrolidine *(**9**)*

*Scheme 2, step e*: LiAlH_4_ (7 mg, 0.19 mmol) was added to a solution of **11** (38 mg, 0.1 mmol) in dry THF (1 mL) at 0 °C. The resulting mixture was stirred for 1h, allowing to warm to rt, whereupon the reaction was quenched with wet ether, dried (Na_2_SO_4_), filtered, and concentrated. The resulting crude residue was purified by flash chromatography (silica gel, n-hexane/EtOAc 8:2) to yield benzyl derivative **9** (10 mg, 30%). IR (film) ν (cm^−1^) 2983, 2942, 1454, 1377, 1307, 1209, 1148, 1086, 1054; ^1^H-NMR (400 MHz) δ = 7.90–7.15 (m, 10H), 4.70 (m, 1H), 4.64 (m, 1H), 3.81 (d, *J* = 13.3 Hz, 1H), 3.55 (d, *J* = 13.3 Hz, 1H), 3.32–3.27 (m, 2H), 3.13 (dd, *J* = 13.5 and 9.0 Hz, 1H), 2.87 (dd, *J* = 10.7 and 5.3 Hz, 1H), 2.68 (d, *J* = 10.7 Hz, 1H), 1.50 (s, 3H),1.28 (s, 3H); ^13^C-NMR (100 MHz) δ = 139.4, 137.0, 133.7, 129.2, 128.3, 128.1, 127.1, 112.4, 84.3, 78.7, 63.4, 57.3, 56.6, 55.1, 27.0, 25.0; HRMS (ESI) C_21_H_25_NO_4_S requires (M+Na) 410.1402; found 410.1397; [α]_D_^20^ = + 20.2 (*c* 1.0, CHCl_3_).

*Scheme 2, step f*: To a solution of **11** (38 mg, 0.1 mmol) in dry THF (1 mL) under argon at rt, was added BH_3_·THF (0.12 mL, 1M in THF). After stirring for 2 h at 40 °C, the reaction was quenched by the addition of water (0.3 mL) and extracted with Et_2_O (3 × 10 mL). The combined organic layers were washed with H_2_O and brine, dried (Na_2_SO_4_), filtered, and concentrated. The resulting crude residue was purified by flash chromatography (silica gel, n-hexane/EtOAc 8:2) to obtain benzyl derivative **9** (5 mg, 15%).

### 4.5. Preparation of (2R,3S,4R)-2-Phenylsulfonylmethyl-3,4-isopropylidenedioxypyrrolidine *(**12**)*

*Scheme 2, step g*: To a solution of pyrrolidine **9** (10 mg, 0.03 mmol) in MeOH (0.5 mL) was hydrogenated in the presence of a catalytic amount of Pd/C with a H_2_ balloon at room temperature for 24 h. The catalyst was filtered through a pad of Celite^®^ and washed with MeOH. After concentrating, compound **12** (6 mg, 80%) was obtained. IR (film) ν (cm^−1^) 3317, 2985, 2936, 1448, 1375, 1306, 1209, 1144, 1083, 1046, 865; ^1^H-NMR (400 MHz) δ = 7.93 (m, 2H), 7.65–7.54 (m, 3H), 4.67 (dd, *J* = 4.4 and 5.3 Hz, 1H), 4.57 (d, *J* = 5.3 Hz, 1H), 3.57 (t, *J*= 6.4 Hz, 1H), 3.13 (d, *J* = 13.2 Hz, 1H), 3.05 (d, *J* = 13.6 Hz, 1H), 2.69 (dd, *J* = 4.4 and 13.2 Hz, 1H), 1.44 (s, 3H), 1.27 (s, 3H); ^13^C-NMR (100 MHz) δ = 139.4, 133.8, 129.3, 128.1, 111.6, 85.0, 84.9, 60.3, 57.2, 51.8, 26.2, 24.1; HRMS (ESI) C_14_H_20_NO_4_S requires (M+H^+^) 298.1113; found 298.1115; [α]_D_^20^ = −13.1 (*c* 1.7, CHCl_3_).

### 4.6. Preparation of (3S,4R)-3,4-Isopropylidenedioxypyrroline-1-oxide *(**13**)*

To a solution of lactol **2** (1.34 g, 8.54 mmol) in dry pyridine (8.6 mL) containing 3 Å activated molecular sieves (pellets, 10 g) was added a solution of NH_2_OSiMe_2_*t*-Bu (1.51 g, 10.25 mmol) in pyridine (8.6 mL) and the mixture was stirred at rt for 16 h. The reaction mixture was cooled to 0 °C, and methanesulfonyl chloride (0.8 mL, 10.25 mmol) was added slowly during 40 min. The reaction was stirred for 2 h at 0 °C, warmed to rt and stirred for 4 h. The mixture was then dilueted with CH_2_Cl_2_ (9 mL), filtered through Celite^®^, and concentrated. The resulting crude residue was purified by flash chromatography (silica gel, CH_2_Cl_2_/EtOAc/MeOH 15/7/1) to give pure nitrone **13** (635 mg, 50%). IR (film) ν (cm^−1^) 3084, 2993, 2980, 1579; ^1^H-NMR (400 MHz) δ = 6.82 (q, *J*= 1.5 Hz, 1H), 5.24 (d, *J* = 6.2, 1H), 4.86 (ddd, *J*= 6.2, 5.1 and 1.5 Hz, 1H), 4.07–3.99 (m, 2H), 1.40 (s, 3H), 1.31 (s, 3H); HRMS (ESI) C_7_H_12_NO_3_ requires (M+H^+^) 158.1785; found 158.0822; [α]_D_^20^ = −26.9 (*c* 1.1, CH_2_Cl_2_).

### 4.7. Preparation of (2R,3S,4R)-2-Phenylsulfonylmethyl-1-hydroxy-3,4-isopropylidenedioxypyrrolidine *(**14**)*

To a stirred solution of MeSO_2_Ph (520 mg, 2.58 mmol) in THF (4.7 mL) was added slowly *n*-BuLi (1.9 mL, 2.58 mmol) and the mixture was reacted at 0 °C for 10 min. Afterwards, the reaction mixture was cooled to −78 °C and stirred for 10 min at this temperature. Then, it was added into a solution of nitrone **13** (320 mg, 2.03 mmol) in THF (6.7 mL) and the mixture was stirred at −78 °C for 30 min and then for 2 h allowing to warm to rt. The reaction was quenched with saturated aqueous solution of NH_4_Cl and the product was extracted with EtOAc (3 × 15 mL). The combined organic layers were washed with brine, dried (Na_2_SO_4_), filtered, and concentrated. The resulting crude residue was purified by flash chromatography (silica gel, n-hexane/EtOAc 1:1) to obtain hydroxylamine **14** (300 mg, 48%). IR (film) ν (cm^−1^) 3428, 3207, 2987, 2925, 2856, 1581, 1442, 1385, 1295, 1131, 1074, 845; ^1^H-NMR (400 MHz) δ = 7.96 (m, 2H), 7.67–7.53 (m, 3H), 6.25 (s, 1H), 4.75 (m, 1H), 4.62 (dd, *J* = 4.2 and 6.0 Hz, 1H), 3.69 (dd, *J* = 6.4 and 14.0 Hz, 1H), 3.44 (m, 1H), 3.37 (m, 1H), 3.25 (dd, *J* = 6.8 and 14.0 Hz, 1H), 3.12 (dd, *J* = 4.4 and 12.4 Hz, 1H), 1.43 (s, 3H), 1.27 (s, 3H); ^13^C-NMR (100 MHz) δ = 139.4, 133.9, 129.3, 128.1, 113.7, 82.2, 77.2, 67.3, 62.3, 54.1, 26.7, 24.7; HRMS (ESI) C_14_H_19_NO_5_S requires (M + Na) 336.0882; found 336.0877; [α]_D_^20^ = −20.8 (*c* 2.2, CHCl_3_).

### 4.8. Preparation of (2S,3S,4R)-2-Phenylsulfonylmethyl-3,4-isopropylidenedioxypyrrolidine *(**6**)*

*Scheme 3, step c*: A stirred solution of hydroxylamine **14** (50 mg, 0.16 mmol) in a 12:1 solution of HCl/MeOH (1 mL) was hydrogenated in the presence of a catalytic amount of Pd (OH)_2_/C, at a hydrogen pressure of 4 atm for 48 h. The catalyst was filtered through a pad of Celite,^®^ washed with MeOH and concentrated. The product was purified by flash chromatography (silica gel, n-hexane/EtOAc 1:1) to provide pyrrolidine **6** (13 mg, 27%). IR (film) ν (cm^−1^) 3000, 2936, 1447, 1381, 1308, 1148, 1086, 650 cm^−1^; ^1^H-NMR (400 MHz) δ = 7.93 (m, 2H), 7.69–7.50 (m, 3H), 4.67 (dd, *J* = 4.0 and 5.3 Hz, 1H), 4.54 (dd, *J* = 4.0 and 5.3 Hz, 1H), 3.57 (dd, *J* = 5.0 and 14.0 Hz, 1H), 3.36 (dd, *J* = 7.0 and 14.0 Hz, 1H), 3.22 (m, 1H), 3.13 (d, *J* = 12.7 Hz, 1H), 2.69 (dd, *J* = 4.0 and 12.7 Hz, 1H), 2.20 (s, 1H), 1.41 (s, 3H) and 1.25 (s, 3H); ^13^C-NMR (100 MHz) δ = 139.7, 133.7, 129.2, 127.2, 111.1, 81.0, 80.8, 57.1, 55.9, 52.6, 25.7, 24.0; HRMS (ESI) C_14_H_20_NO_4_S requires (M+H^+^) 298.1113; found 298.1128; [α]_D_^20^ = −37.3 (*c* 0.5, CHCl_3_).

*(Scheme 3, step d)*: A stirred solution of hydroxylamine **14** (66 mg, 0.21 mmol) in 1 mL of MeOH was hydrogenated in the presence of a catalytic amount of Pd/C with a H_2_ balloon at room temperature for 24 h. The catalyst was filtered through a pad of Celite,^®^ washed with MeOH and concentrated. The product was purified by flash chromatography (silica gel, n-hexane/EtOAc 1:1) to provide pyrrolidine **6** (24 mg, 39%). 

### 4.9. Preparation of (2R,3S,4R)-2-Phenylsulfonylmethyl-1-hydroxy-3,4-isopropylidenedioxypyrrolidine *(**15**)*

*(Scheme 3, step e)*: To a stirred solution of hydroxylamine **14** (32.1 mg, 0.10 mmol) in a 2:1 solution of EtOH /saturated aqueous NH_4_Cl (18.5 mL), powdered indium (14 g, 0.12 mmol) was added and the mixture was heated under reflux. After 24 h the reaction mixture was cooled, filtered through Celite,^®^ and concentrated under reduced pressure. A saturated aqueous Na_2_CO_3_ solution (5 mL) was then added, and the product was extracted with EtOAc (3 × 15 mL). The combined organic layers were washed with brine, dried (Na_2_SO_4_), filtered, and concentrated. The resulting crude residue was purified by flash chromatography (silica gel, hexane/EtOAc 1:1) to obtain hydroxylamine **15** (28.8 mg, 92%). IR (film) ν (cm^−1^) 3448, 2985, 2933, 2854, 1448, 1383, 1306, 1085, 856; ^1^H-NMR (400 MHz) δ = 7.97 (m, 2H), 7.67–7.52 (m, 3H), 6.27 (s, 1H), 4.60 (m, 2H), 3.70 (dd, *J* = 8.4 and 14.o Hz, 1H), 3.50–3.38 (m, 2H), 3.05 (m, 1H), 2.70 (dd, *J* = 4.4 and 11.0 Hz, 1H), 1.37 (s, 3H), 1.22 (s, 3H); ^13^C-NMR (100 MHz) δ = 139.9, 134.1, 129.5, 128.3, 111.1, 77.2, 76.4, 65.9, 62.2, 54.5, 25.9, 24.4; HRMS (ESI) C_14_H_19_NO_5_S requires (M + Na) 336.0882; found 336.0895; [α]_D_^20^ = −10.4 (*c* 1.3, CHCl_3_).

*Scheme 3, step f*: To a stirred solution of hydroxylamine **14** (69 mg, 0.22 mmol) in a solution of MeOH/NH_4_Cl_sat_ (3.4 /5 mL), powdered Zn (29 mg, 0.44 mmol) was added at 20 °C and the mixture was stirred for 6h. The solvent was evaporated under vacuum and a saturated aqueous solution of Na_2_CO_3_ (1.5 mL) was added. The mixture was extracted with EtOAc (3 × 15 mL) and the combined organic layers were washed with brine, dried (Na_2_SO_4_), filtered, and concentrated. The resulting crude residue was purified by flash chromatography (silica gel, n-hexane/EtOAc 1:1) to obtain hydroxylamine **15** (34 mg, 50%).

### 4.10. Preparation of (2R,3S,4R)-2-Phenylsulfonylmethyl-3,4-isopropylidenedioxypyrrolidine *(**12**)*

*Scheme 3, step g, Table 1, entry 8, as example*: To a stirred solution of hydroxylamine **14** (852 mg, 2.58 mmol), in methanol (37 mL), a saturated solution of NH_4_Cl (56 mL), powdered Zn (712 mg, 10.9 mmol) and a catalytic amount of indium dust (20 mg) were added at 20 °C. The mixture was stirred for 6h. The solvent was evaporated under vacuum and a saturated aqueous solution of Na_2_CO_3_ (5 mL) was added. The mixture was extracted with EtOAc (3 × 15 mL). The combined organic layers were washed with brine, dried (Na_2_SO_4_), filtered, and concentrated. The resulting crude residue was purified by flash chromatography (silica gel, n-hexane/EtOAc 1:1) to obtain pyrrolidines **12** (248 mg, 35%) and **6** (13 mg, 2%).

### 4.11. (2S,1´R)-2-[1´-Phenyl-2´-nitroethyl]-cyclohexanone

To a suspension of catalyst **6** (10 mg, 15%) and 0.438 mL (4.40 mmol) of cyclohexanone in 3.5 mL of DMSO was added 33 mg (0.22 mmol) of trans-β nitrostyrene. The resulting mixture was allowed to stir at room temperature for 16 h, whereupon the reaction was quenched with saturated aqueous ammonium chloride (2 mL) and the aqueous layers were extracted with ethyl acetate. The combined organic layers were dried over Na_2_SO_4_, filtered and evaporated in vacuo and the resulting residue was purified by flash column chromatography using (hexane/EtOAc, 9/1) to give the title compound, as a white solid (11mg, 20%). ^1^H-NMR (400 MHz) δ 7.34–7.16 (m, 5H, Ph), 4.93 (dd, *J* = 12.5 and 4.5 Hz, 1H, CH_2_), 4.60 (dd, *J* = 12.5 and 9.9 Hz, 1H, CH_2_), 3.76 (m, 1H, CH), 2.69 (m, 1H, CH), 2.50–1.52 (m, 8H, 4CH_2_). The absolute configuration was established by comparison with reported HPLC data [21]. HPLC: Daicel Chiralpak AD; n-hexane/^i^PrOH: 0.45 mL min^−1^; λ_max_230 nm: t_R_ (minor)-10.8 min; t_R_ (major)-13.1 min.

### 4.12. (2R,1´S)-2-[1´-Phenyl-2´-nitroethyl]-cyclohexanone

To a suspension of catalyst **12** (10 mg, 15%) and 0.438 mL (4.40 mmol) of cyclohexanone in 3.5 mL of CHCl_3_ was added 33 mg (0.22 mmol) of trans-β nitrostyrene. The resulting mixture was allowed to stir at room temperature for 16 h, whereupon the reaction was quenched with saturated aqueous ammonium chloride (2 mL) and the aqueous layers were extracted with ethyl acetate. The combined organic layers were dried over Na_2_SO_4_, filtered and evaporated in vacuo and the resulting residue was purified by flash column chromatography using (n-hexane/EtOAc, 9/1) to give the title compound, as a white solid (22mg, 35%). The absolute configuration was established by comparison with reported HPLC data [21]. HPLC: Daicel Chiralpak AD; n-hexane/^i^PrOH: 0.45 mL min^−1^; λ_max_230 nm: t_R_ (major)-10.5 min; t_R_ (minor)-12.9 min.

## Figures and Tables

a. PhCOCl, Py., 0 °C-r.t., 97%; b. n-BuLi, THF, −78 °C-r.t., 50%; c NH_2_-NH_2_, EtOH, reflux, 0%; d. HCl 6M, reflux, 0%; e. LiAlH_4_, THF, 0 °C-r.t., 30%; f. BH_3_·THF, THF, 0 °C-40 °C, 15%; g. H_2_, Pd/C, MeOH, r.t., 80%.

a. 1. NH_2_OSiMe_2_t-Bu, Py., r.t., 15h.; 2. MsCl, 0 °C, 2h, 50%; b. MeSO_2_Ph, n-BuLi, THF, −78 °C, 48%; c. H_2_, Pd(OH)_2_/C, HCl / MeOH, 4atm, 27%; d. H_2_, Pd/C, MeOH, 39%; e. In, NH_4_Cl/EtOH, reflux, 24h, 92%; f. Zn, NH_4_Cl/MeOH, reflux, 2h, 50%; g. In(cat), Zn, NH_4_Cl/MeOH reflux or r.t., see Table 1.

**Table 1:**
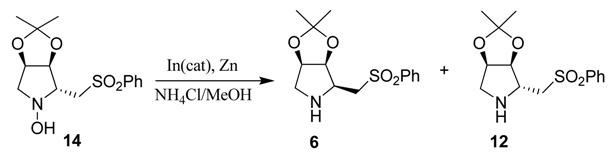
Reduction conditions of hydroxylamine **14** to pyrrolidines **6** and **12**.

Entry	Zn Equiv.	T^a^	t(h)	η (%)	Ratio 6/12
**1**	6	Reflux	1	15	40/60
**2**	4	Reflux	2	10	36/64
**3**	4	Reflux	1	15	30/70
**4**	4	Reflux	0.5	ND^a^	ND^a^
**5**	2	Reflux	1	ND^a^	ND^a^
**6**	2	Reflux	1.5	10	40/60
**7**	2	Reflux	2	20	15/85
**8**	4	r.t.	5.5	40	5/95
**9**	3	r.t.	4	8	45/55
**10**	2	r.t.	4	ND^a^	ND^a^

^a^ Not determined.

**Table 2:**
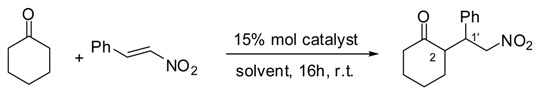
Solvent effects on the asymmetric Michael addition of cyclohexanone to *trans*-β-nitrostyrene with catalysts **6** and **12**.

Entry^[a]^	Solv.	Catalyst	Yield [%]^[b]^	d.r.[%] ^[c]^	ee[%]^[d]^	Conf.
**1**	CHCl_3_	**6**	-	-	-	-
**2**	CHCl_3_	**12**	35	>95	38	*2R, 1’S*
**3**	DMSO	**6**	20	>95	69	*2S, 1’R*
**4**	DMSO	**12**	-	-	-	-

[a] For experimental conditions see the Experimental section. [b] Yield of the isolated product. [c] Determined by ^1^H-NMR spectroscopic analysis. [d] Determined by chiral high-performance liquid chromatography (HPLC) analysis (Daicel Chiralpak AD, 25 cm/4.6 mm/5 μ).
